# Supplementary material for: The vortex-driven dynamics of droplets within droplets
Source: Nat Commun. 2021 Jan 4;12:82. doi: 10.1038/s41467-020-20364-0 (PMC7782531; doi:10.1038/s41467-020-20364-0)
Supplement: Supplementary file 1 — Supplementary Information [file 41467_2020_20364_MOESM1_ESM.pdf]

The vortex-driven dynamics of droplets within droplets
Supplementary Information

A. Tiribocchi,[1,2] A. Montessori,[2] M. Lauricella,[2] F. Bonaccorso,[1,2] S. Succi,[1,2,3] S. Aime,[3,4] M. Milani,[5] and D. Weitz[3,6]

[1] *Center for Life Nano Science@La Sapienza, Istituto Italiano di Tecnologia, 00161 Roma, Italy*
[2] *Istituto per le Applicazioni del Calcolo CNR, via dei Taurini 19, Rome, Italy*
[3] *Institute for Applied Computational Science, John A. Paulson School of Engineering and Applied Sciences, Harvard University, Cambridge, Massachusetts 02138, USA*
[4] *Matière Molle et Chimie, Ecole SupÃ©rieure de Physique et Chimie Industrielles, 75005 Paris, France*
[5] *Università degli Studi di Milano, via Celoria 16, 20133, Milano, Italy*
[6] *Department of Physics, Harvard University, Cambridge, Massachusetts 02138, USA*

SUPPLEMENTARY NOTE 1: NUMERICAL DETAILS

Here we provide further details about the numerical method and the simulation parameters.

The equations for the order parameter ϕ_i (Equation 2 of the main text) and the Navier-Stokes equation (Equation 3 of the main text) are solved by using a hybrid lattice Boltzmann (LB) method [1], in which Equation 2 is integrated via a finite-difference predictor-corrector algorithm and Equation 3 via a standard LB approach.

As reported in the main text, simulations are peformed on a rectangular lattice with size ratio $\Gamma = \frac{L_z}{L_y}$ ranging from 0.16 to 0.22. More specifically, $\Gamma = 0.167$ ($L_y = 600$, $L_z = 100$) for the core-free droplet, $\Gamma = 0.2$ ($L_y = 600$, $L_z = 120$) for the single-core emulsion and $\Gamma = 0.21$ ($L_y = 800$, $L_z = 170$) for the two-core and higher complex emulsions. Periodic boundary conditions are set along the y-axis and two flat walls along the z-axis, placed at $z = 0$ and $z = L_z$. Here no-slip conditions hold for the velocity field (i.e. $v_z(z = 0, z = L_z) = 0$) and neutral wetting for the order parameters ϕ_i. The latter ones are achieved by setting

$$\left.\frac{\partial \mu_i}{\partial z}\right|_{z=0,z=L_z} = 0 \qquad (1)$$

$$\left.\frac{\partial \nabla^2 \phi_i}{\partial z}\right|_{z=0,z=L_z} = 0. \qquad (2)$$

The first one guarantess density conservation (no mass flux through the walls) while the second one imposes the wetting to be neutral.

Like in previous works [2, 3], the pressure gradient Δp producing the Poiseulle flow is modeled through a body force (force per unit density) added to the collision operator of the LB equation at each lattice node.

Thermodynamic parameters have been chosen as follows: $a = 0.07$, $k = 0.1$, $M = 0.1$ and $\epsilon = 0.05$ (a value larger than 0.005 is enough to prevent droplet merging). These values fix the surface tension and the interface width to $\sigma = \sqrt{\frac{8ak}{9}} \simeq 0.08$ and $\xi = 2\sqrt{\frac{2k}{a}} \simeq 3-4$, respectively. Also, the dynamic viscosity η of both fluid components is set equal to 5/3. Such approximation, retained for simplicity, may be relaxed by letting η depends on ϕ [4, 5]. Lattice spacing and integration time-step have been kept fixed to $\Delta x = 1$ and $\Delta t = 1$, while droplet radii are chosen as follows: $R = 30$ for the core-free droplet, $R_{\mathrm{i}} = 15$ and $R_{\mathrm{O}} = 30$ for a single-core emulsion, and $R_{\mathrm{i}} = 17$ and $R_{\mathrm{O}} = 56$ for emulsions containing more than one core. Here R_{i} is the radius of the cores while R_{O} is the one of the surrounding shell.

SUPPLEMENTARY NOTE 2: VELOCITY PROFILE UNDER POISEUILLE FLOW

In Supplementary Figure 1 we report, for example, the typical steady-state velocity profile observed in a two-core emulsion for different values of the pressure gradient. They are averaged over space and time, i.e. the channel length and approximately 3×10^5 time steps at the steady state. The curves are compatible with a parabolic profile expected in an isotropic fluid with the same viscosity, and remain essentially unaltered for the other multi-core emulsions considered in this work.

However, substantial modifications occur when instantaneous configurations are considered. In Supplementary Figure 2 we show, for instance, the instantaneous velocity profile observed in core-free (a), one-core (b), two-core (c) and three-core (d) emulsions calculated along a cross section of the channel where internal cores temporarily accumulate. While in (a) the parabolic profile is only weakly disturbed by the droplet interface, in (b)-(d) it is significantly modified by local bumps and dips caused by internal cores. Such distortions wash out when these profiles are averaged over space and time.

SUPPLEMENTARY NOTE 3: STRUCTURE OF THE VELOCITY FIELD IN FOUR, FIVE AND SIX-CORE EMULSIONS

In Supplementary Figure 3 we show the typical velocity field observed in multiple emulsions containing (a) four,

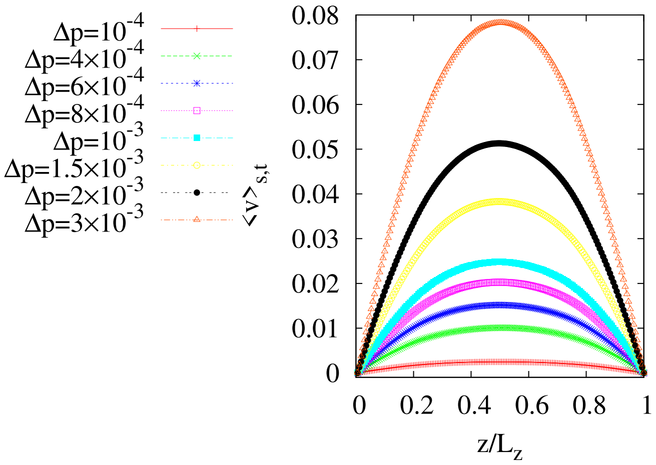

Supplementary Figure 1. Averaged Poiseuille profile. This plot shows the typical steady-state velocity profile for different values of pressure gradient Δp in a two-core emulsion. Here $\langle v \rangle$ is averaged over space and time.

(b) five and (c) six cores. In all cases, the interior structure of the field exhibits significant deviations (heavier as the number of cores increases) from the double-vortex pattern of a core-free emulsion.

As discussed in the paper, under Poiseuille flow a four-core emulsion, originally designed as in Fig.2d of the main text, only temporarily survives in a state of the form $\langle 1, 2, 3|4 \rangle$ (Supplementary Figure 3a), since the effective area fraction occupied by three cores is larger than 0.35 in half emulsion. This causes a crossing of a drop (3 in Supplementary Figure 3a) driven a heavy flux pushing it downwards, thus leading to the long-lived nonequilibrium state $\langle 1, 2|3, 4 \rangle$ (see Fig.3h of the main text). In this state, couples of drops display a planetary-like motion within each half of the emulsion and no further crossing occurs.

Increasing the number of inner drops, such as in five and six-core systems (Supplementary Figure 3b,c), favours multiple crossings between the two regions of the emulsions, a process generally driven by a pre-chaotic flows resulting from the complex coupling between velocity and phase field. This is why only short-lived states are observed in these systems.

SUPPLEMENTARY REFERENCES

[1] L. N. Carenza, G. Gonnella, A. Lamura, G. Negro, A. Tiribocchi, Lattice Boltzmann Methods and Active Fluids, Eur. Phys. Journ. E **42**, 81 (2019).

[2] M. Foglino, A. N. Morozov, O. Henrich, D. Marenduzzo, Flow of Deformable Droplets: Discontinuous Shear Thinning and Velocity Oscillations, Phys. Rev. Lett. **119**, 208002 (2017).

[3] M. Foglino, A. Morozov, D. Marenduzzo, Rheology and microrheology of deformable droplet suspensions, Soft Matter **14**, 9361-9367 (2018).

[4] K. Langaas, J. M. Yeomans, Lattice Boltzmann simulation of a binary fluid with different phase viscosities and its application to fingering in two dimensions, Eur. Phys. Journ. B **15**, 133-141 (2000)

[5] E. Tjhung, A. Tiribocchi, D. Marenduzzo, M. E. Cates, A minimal physical model captures the shapes of crawling cells, Nat. Comm. **6**, 5420 (2015).

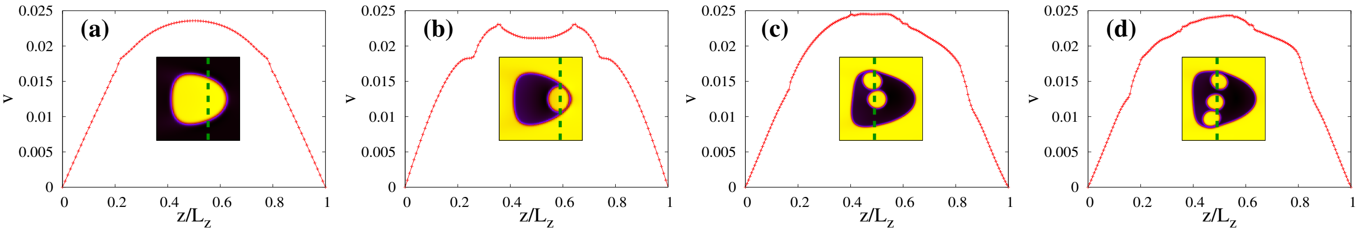

Supplementary Figure 2. Instantaneous Poiseuille profile. These plots show the instantaneous velocity profile of a core-free (a), one-core (b), two-core (c) and three-core (d) emulsion computed along the cross section of the channel, indicated by the dotted green line. Insets show the corresponding configuration of the emulsion.

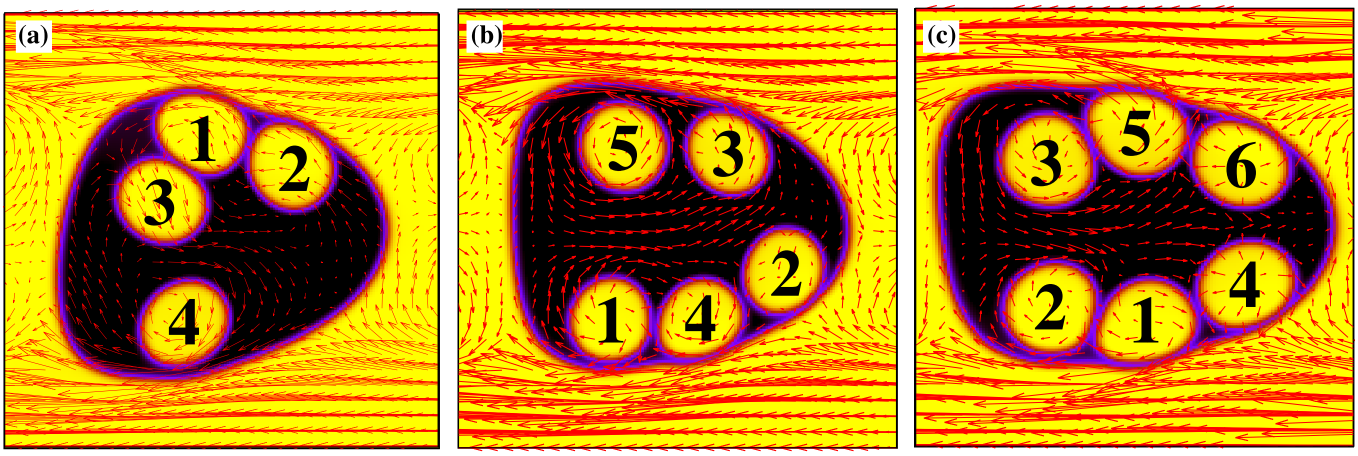

Supplementary Figure 3. Velocity field. Characteristic structures of the velocity field in (a) four-core (b) five-core and (c) six-core emulsions computed with respect to the external droplet frame. The typical double eddy structure observed in a core-free emulsion undergoes significant distortions as the number of cores increases. Panel (a) shows the flow field observed during the crossing of droplet 3 from the top towards the bottom of the emulsion (see also Fig.9 of the main text). A transition from the state $\langle 1,2,3|4\rangle$ to the state $\langle 1,2|3,4\rangle$ occurs. Panels (b) and (c) show two short-lived states of the form $\langle 3,5|1,2,4\rangle$ and $\langle 3,5,6|1,2,4\rangle$, only temporarily surviving due to multiple crossings.
