# Supplementary material for: The vortex-driven dynamics of droplets within droplets
Source: Nat Commun. 2021 Jan 4;12:82. doi: 10.1038/s41467-020-20364-0 (PMC7782531; doi:10.1038/s41467-020-20364-0)
Supplement: Supplementary file 3 — Description of Additional Supplementary Files [file 41467_2020_20364_MOESM3_ESM.pdf]

Description of Additional Supplementary Files

Supplementary Movie 1: This movie shows the dynamics of a core-free emulsion under Poiseuille flow at Re ' 3 and Ca ' 0.85 (see Fig.1 of the main text).

Supplementary Movie 2: This movie shows the dynamics of a one-core droplet under Poiseuille flow at Re ' 1.2 and Ca ' 0.35 (Fig.3b of the main text).

Supplementary Movie 3: This movie shows the dynamics of a two-core droplet (Ac $\sim$ 0.18) under Poiseuille flow at Re ' 3 and Ca ' 0.85 (Fig.3c of the main text). Here the cores are confined within the upper part of the emulsion and exhibit a planetary-like dynamics.

Supplementary Movie 4: This movie shows the dynamics of a two-core droplet (Ac $\sim$ 0.18) under Poiseuille flow at Re ' 3 and Ca ' 0.85 (Fig.3d of the main text). The two cores remain separately confined within the top and the bottom of the emulsion.

Supplementary Movie 5: This movie shows the dynamics of a three-core droplet (Ac $\sim$ 0.27) under Poiseuille flow at Re ' 3 and Ca ' 0.85 (Fig.3e of the main text). Here two cores exhibit a planetary-like dynamics in the bottom of the emulsion while the other core remains locked in the upper part and moves along approximately circular trajectories.

Supplementary Movie 6: This movie shows the dynamics of a three-core droplet (Ac $\sim$ 0.27) under Poiseuille flow at Re ' 3 and Ca ' 0.85 (Fig.3f of the main text). The three cores display a complex three-body periodic motion, confined within half of the emulsion.

Supplementary Movie 7: This movie shows the dynamics of a four-core droplet (Ac $\sim$ 0.37) under Poiseuille flow at Re ' 3 and Ca ' 0.85 (Fig.3g and Fig.3e of the main text). Here a three-core state survives only for a short period of time, since one of its cores crosses from the top towards the bottom of the emulsion. This yields to a long-lived state in which couples of cores exhibit a planetary motion within two separate regions of the emulsion.

Supplementary Movie 8: This movie shows the dynamics of a four-core droplet (Ac $\sim$ 0.37) under Poiseuille flow, in which the cores are initially located in the lower part of the emulsion. Here Re ' 3

and Ca ' 0.85. The two inner drops on top move towards the leading edge of the emulsion and then are captured by the fluid vortex in un upper region. A long-lived state, in which periodic motion of the cores occurs in two different parts of the emulsion, emerges anew.

Supplementary Movie 9: This movie shows the dynamics of a five-core droplet (Ac $\sim$ 0.46) under Poiseuille flow at Re ' 3 and Ca ' 0.85 (Fig.3ij of the main text). Only short living states are observed, since multiple crossings occur within the emulsion.

Supplementary Movie 10: This movie shows the dynamics of a six-core droplet (Ac $\sim$ 0.55) under Poiseuille flow at Re ' 3 and Ca ' 0.85 (Fig.3k-l of the main text).

Supplementary Movie 11: This movie shows the dynamics of a four-core droplet with Ac $\sim$ 0.2 under Poiseuille flow at Re ' 3 and Ca ' 0.85. Here the inner drops are initially located in the lower part of the emulsion. A nonequilibrium state of the form h0|1, 2, 3, 4i occurs at late times.

Supplementary Movie 12: This movie shows the dynamics of a four-core droplet with Ac $\sim$ 0.2 under Poiseuille flow at Re ' 3 and Ca ' 0.85. Here the inner drops are initially located symmetrically as in Fig.2d of the main text. A nonequilibrium state of the form h1, 3|2, 4i occurs at late times.
